# A Systems-Level Analysis of Total-Body PET Data Reveals Complex Skeletal Metabolism Networks *in vivo*

**DOI:** 10.3389/fmed.2021.740615

**Published:** 2021-09-20

**Authors:** Karla J. Suchacki, Carlos J. Alcaide-Corral, Samah Nimale, Mark G. Macaskill, Roland H. Stimson, Colin Farquharson, Tom C. Freeman, Adriana A. S. Tavares

**Affiliations:** ^1^University/British Heart Foundation (BHF) Centre for Cardiovascular Science, The Queen's Medical Research Institute, University of Edinburgh, Edinburgh, United Kingdom; ^2^Edinburgh Imaging, University of Edinburgh, Edinburgh, United Kingdom; ^3^The Roslin Institute, The Royal (Dick) School of Veterinary Studies (RDSVS), Easter Bush Campus, University of Edinburgh, Edinburgh, United Kingdom

**Keywords:** bone, positron emission tomography, metabolism, system biology, network analysis

## Abstract

Bone is now regarded to be a key regulator of a number of metabolic processes, in addition to the regulation of mineral metabolism. However, our understanding of complex bone metabolic interactions at a systems level remains rudimentary. *in vitro* molecular biology and bioinformatics approaches have frequently been used to understand the mechanistic changes underlying disease at the cell level, however, these approaches lack the capability to interrogate dynamic multi-bone metabolic interactions *in vivo*. Here we present a novel and integrative approach to understand complex bone metabolic interactions *in vivo* using total-body positron emission tomography (PET) network analysis of murine ^18^F-FDG scans, as a biomarker of glucose metabolism in bones. In this report we show that different bones within the skeleton have a unique glucose metabolism and form a complex metabolic network, which could not be identified using single tissue simplistic PET standard uptake values analysis. The application of our approach could reveal new physiological and pathological tissue interactions beyond skeletal metabolism, due to PET radiotracers diversity and the advent of clinical total-body PET systems.

## Introduction

The availability of large *in vitro* cell and tissue omic datasets and bioinformatic tools have equipped researchers to understand molecular processes that cause disease, and identify and develop new therapeutics (1). However, novel systems approaches are needed to understand complex *in vivo* physiological and pathological interactions at multi-tissue level. Positron emission tomography (PET) imaging allows for the non-invasive *in vivo* investigation of signalling pathways owing to the radiotracer principle and total-body dynamic PET lends itself to deciphering complex biological processes and interactions (2–6), such as those found associated with the skeletal system. Here we present an integrative approach to understand complex tissue interactions *in vivo* using total-body PET network analysis that is directly applicable to emerging clinical total-body dynamic imaging. We initially focused on the skeletal system as it provides an ideal model for analysing complex interactions. The skeleton serves multiple functions *in vivo* such as organ protection, allowing for weight-bearing motion, providing a niche for haematopoiesis and has recently emerged to have major endocrine functions, for example by the bone-specific protein, osteocalcin (7–10). Bone and cartilage are significant sites of glucose uptake in mice and humans (9, 11, 12). However, it remains unclear if different bones within the skeleton have specialised roles in glucose metabolism. Here, we aim to explore whether glucose metabolism in different bones are associated with one another *in vivo* using ^18^F-fluorodeoxyglucose (^18^F-FDG) dynamic total-body PET.

## Methods

### Animals and Study Design

**Figure 1:**
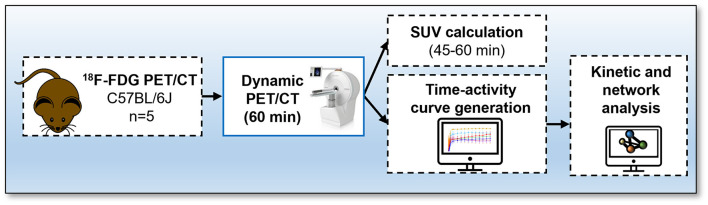
Protocol for ^18^F-fluorodeoxyglucose (FDG) PET/CT. Mice received an intravenous bolus injection *via* tail-vein of ^18^F-FDG and immediately underwent a 60-min total-body emission scan. A CT scan was conducted at the end of each PET scan. Time activity-curves and standard uptake values were calculated and network analysis was performed to visualise interactions between bones using the Pearson correlation values.

Studies were done in compliance with all relevant ethical regulations under project licences granted by the UK Home Office, and were approved by the University of Edinburgh Animal Welfare and Ethical Review Board. Male 13-week-old C57BL/6JCrl (*n* = 5) mice were housed at 22–23°C on a 12 h light/dark cycle with free access to water and food. Animals were fasted for 4 h prior to start of dynamic PET/CT acquisition. The experimental design is outlined in Figure 1.

### Imaging Data Acquisition and Reconstruction

Prior to PET/CT imaging, mice were weighed, anesthetised with a pre-set with a mixture of 0.5/0.5 L/min of oxygen/nitrous oxide and 3% isoflurane and transferred to the microPET/CT scanner (nanoPET/CT, Mediso, Hungary). General anaesthesia was maintained throughout the duration of the PET/CT study (0.5/0.5 L/min of oxygen/nitrous oxide and 2% isoflurane), and vital signs, including temperature and respiration rate, were monitored during the experiments. Animals received a tail vein intravenous bolus injection of ^18^F-fluorodeoxyglucose (^18^F-FDG, 15.08 ± 5.87 MBq, mean ± SD; Group 2). ^18^F-FDG was produced in-house (Edinburgh Imaging) using standard methods of radiolabelling (13).

Immediately following radiotracer administration, animals underwent a total-body emission scan followed by a CT scan (semi-circular full trajectory, maximum field of view, 360 projections, 50 kVp, 300 ms and 1:4 binning). Collected PET images underwent attenuation correction using the CT data. PET images were reconstructed between 0 and 60 min into 6 x 30, 3 x 60, 2 x 120, and 10 x 300 s frames using Mediso's iterative Tetra-tomo 3D reconstruction algorithm and the following settings: four iterations, six subsets, full detector model, low regularisation, spike filter on, voxel size 0.4 mm and 400-600 keV energy window. PET data were corrected for random coincidences, scatter, and attenuation.

### Image Processing and Standard Uptake Value Calculation

Reconstructed images were analysed using PMOD 3.7 software (PMOD Technologies, Switzerland). Volumes of interest (VOI) were drawn around the tibiae, femurs, humeria, radius and ulnas (forearm), spine, sternum and skull. To distinguish bone tissue from bone marrow and surrounding tissues, the VOIs were segmented using previously defined Hounsfield Units, HU, (332-50000) generated using HU obtained from the acquisition of a CT tissue equivalent material (TEM) phantom (CIRS, model 091) and mouse CT scans, as we have previously reported (9). All dynamic PET data were then corrected for time delays between start of the scan and injection of radiotracer. Time activity-curves were generated and standard uptake values (SUVs) were calculated by normalising radioactive concentration in VOI for the injected dose and the animal weight. To estimate the bone uptake at equilibrium, SUV averages were taken from three PET frames between 45 and 60 min post-radiotracer administration. The CT HU were extracted from the VOI of the tibiae, femurs, humeria, forearm spine, sternum, and skull.

### Network Analysis of Total-Body PET Data

Non-decay corrected dynamic total-body PET data was summarised into a table with rows representing average ^18^F-FDG signal from individual bones for all mice and columns the time of the recording. The file was saved as a comma separated variable (.csv) file. This was loaded in the network analysis tool Graphia (https://graphia.app/) (14). Pearson correlations were calculated and a relationship matrix graph was constructed by performing an all versus all comparison of the ^18^F-FDG signal profiles from each bone (correlation cut off value of R > 0.7 and k-nearest neighbours, kNN, of 3). By minimising the number of edges the structure of the relationship between tissue-accumulation profiles are revealed, as reflected by graph's structure and edge weights, where the nodes represent each bone and edges represent correlations above the selected threshold, where the threshold value was set to maintain the number of nodes in the network hence all available data.

### Statistical Analysis, Data Presentation, and Reproducibility

^18^F-FDG SUV averages were analysed for normal distribution using the Shapiro-Wilk normality test. Simple multiple linear regression was conducted to assess CT and SUV correlations. Data are represented as the average ± SEM, unless otherwise stated in the results section. All statistical analysis was performed using Prism 8 (GraphPad v8, USA). Mouse cartoon networks were created with BioRender.com.

## Results

### Murine Bone Density to Energy Metabolism Quotient Diversity Identified by Whole-Body PET/CT Analysis

**Figure 2:**
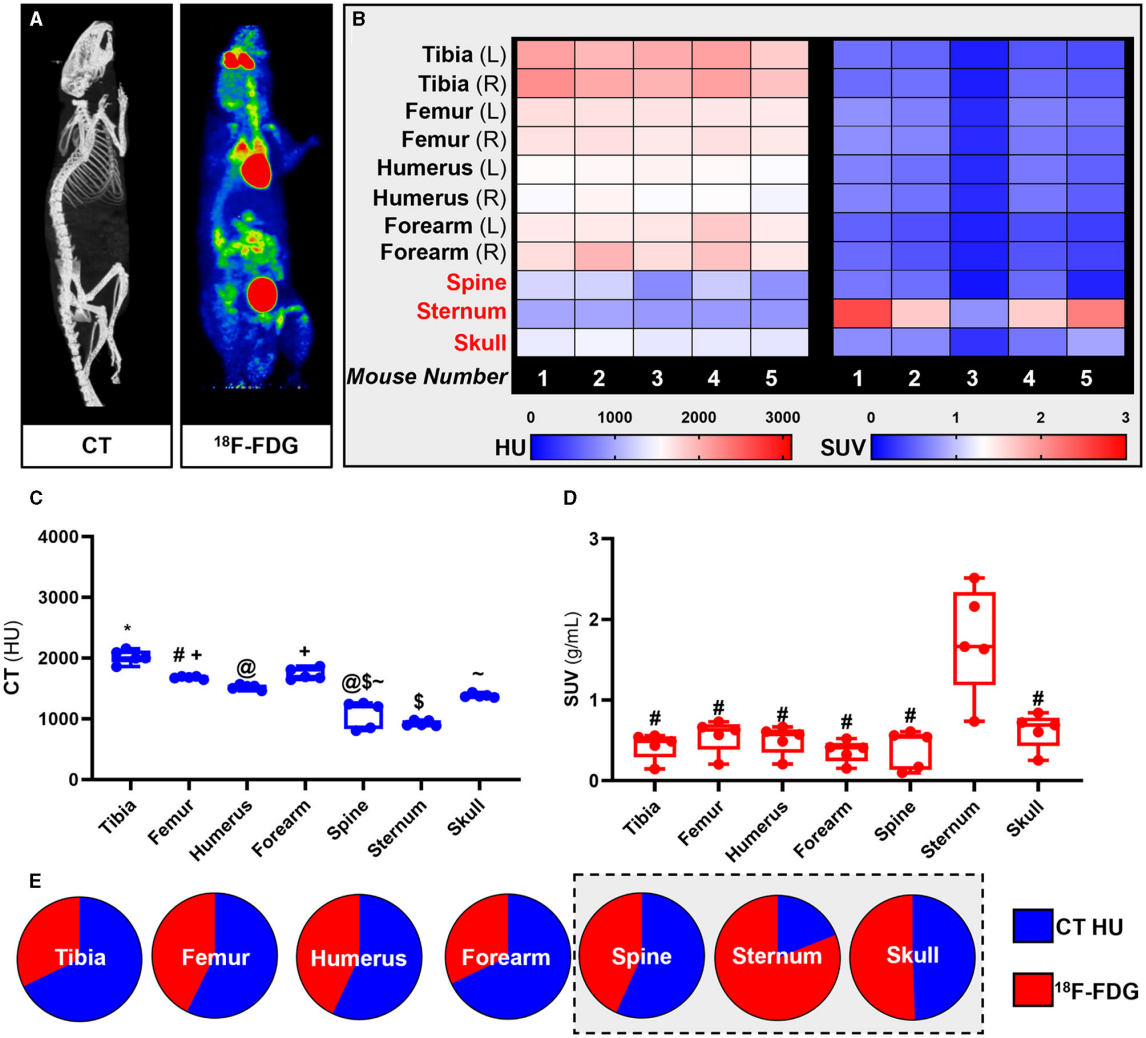
Site-specific metabolic differences in bones identified by whole-body PET/CT analysis. **(A)** Representative maximum intensity projection images of CT and PET data following intravenous administration of ^18^F-FDG. **(B-D)** Hounsfield units (HU) and standard uptake values (SUV) of ^18^F-FDG. SUV was calculated by averaging mouse dynamic PET time-activity curves between 45 and 60 min post-injection into a single static data point for each bone. **(E)** Bone density to energy metabolism quotient. SUV uptake and HU per bone calculated as a percentage of total ^18^F-FDG and HU, respectively, with axial VOIs (sternum, spine, and skull) highlighted in grey. PET SUV percentages were calculated relative to all SUV's measured in the different bones and then plotted with CT HU percentages calculated relative to all CT HU measured in different bones. Heatmaps are measured SUV and HU of five individual mice. Appendicular VOIs (tibia, femur, humerus, and forearm) are highlighted by black text and axial VOIs (sternum, spine, and skull) are highlighted in red text. Box-and-whisker plots; boxes indicate the 25th and 75th percentiles; whiskers display the range; and horizontal lines in each box represent the median. Significant differences were determined by a one-way ANOVA with multiple comparisons. Different symbols above the error bar show significant difference at *P* < 0.05 **(C)**. # indicates different from sternum at *P* < 0.05 **(D)**.

^18^F-FDG PET imaging has been extensively used for quantification of glucose metabolism *in vivo*. Using whole-body ^18^F-FDG PET/CT data (Figure 2A) we extracted the standardised uptake values (SUV) and Hounsfield Units (HU) from the appendicular [tibia, femur, humerus, ulna, and radius (forearm)] and the axial (spine, sternum, and skull) skeleton (Figures 2B–E).

Our PET results showed that overall ^18^F-FDG uptake in the skeleton was bone specific and un-related to bone density measured by quantitative computed tomography (qCT). Measured SUVs in the axial skeleton were higher than in the appendicular skeleton while measured HU from qCT showed higher mineral density in the appendicular skeleton than in the axial skeleton. These findings were congruent throughout the analysis at individual subject-level (heat maps, Figure 2B), group averages statistical analysis (Box-and-whisker plots, Figures 2C,D) and relative fraction analysis (pie-charts, Figure 2E).

### Murine Bone Energy Metabolism Network Identified by Dynamic Total-Body PET/CT Analysis Shows Spine Density Has Strongest Dependence on Glucose Metabolism

**Figure 3:**
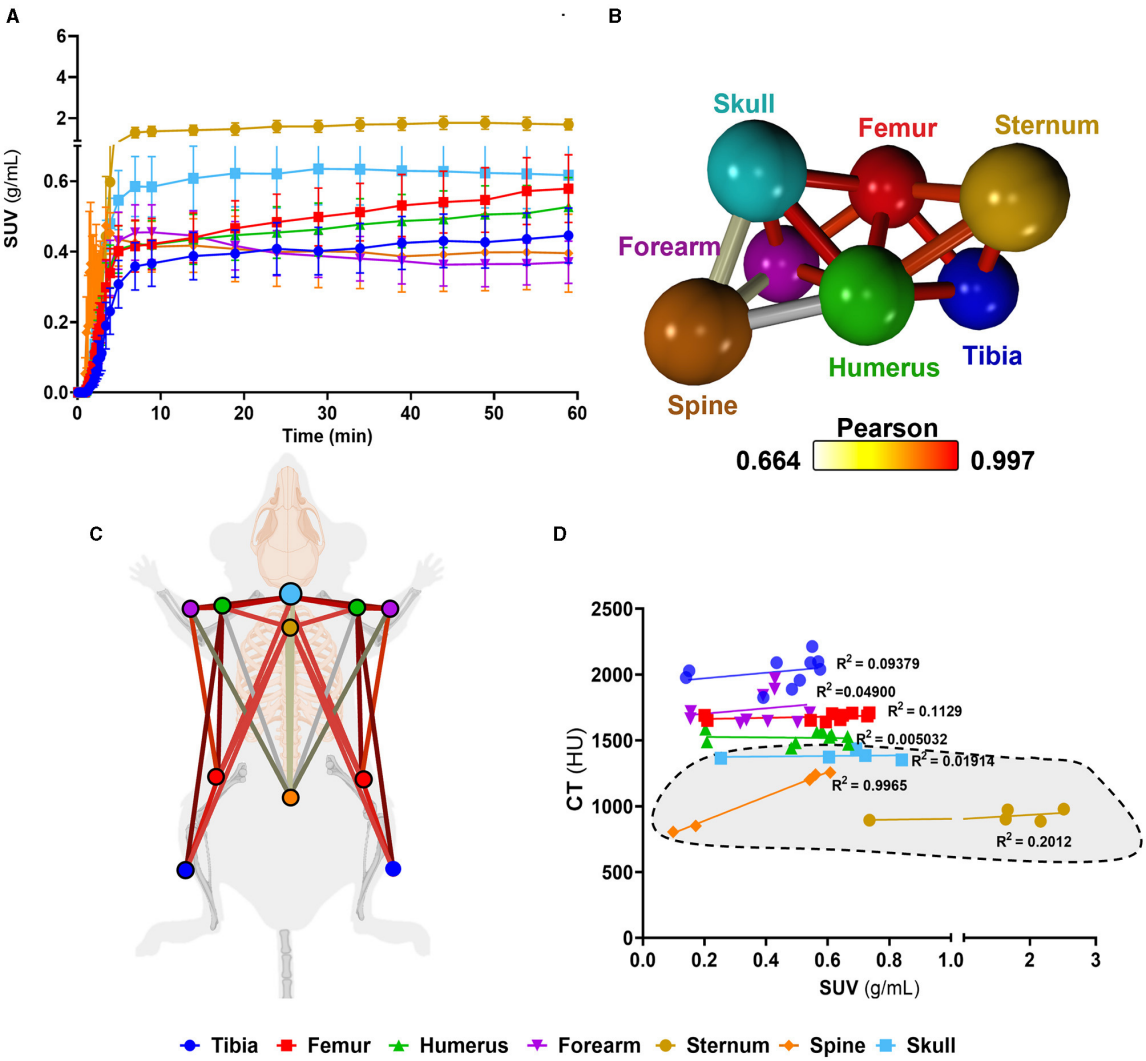
Complex bone metabolic networks identified by innovative dynamic total-body PET network analysis. **(A)** Time-activity curves expressed as standard uptake values (SUV) ^18^F-FDG (*n* = 5). **(B)** Functional network analysis of ^18^F-FDG time-activity curves whereby nodes represent the individual skeletal bones and the edges demote the Pearson correlation value between the nodes (k-nearest neighbours, kNN, of 3). **(C)** Pictorial representation of skeletal networks identified using the functional network analysis of time-activity curves of ^18^F-FDG, nodes are colour coded to represent each bone and the conneting lines demote the Pearson correlation value between the nodes. **(D)** Correlation between computed tomography (CT) Hounsfield units (HU) and SUV from ^18^F-FDG average between 45 and 60 min. Data are presented as the mean ± SEM, *n* = 5. Simple linear regression R^2^ values for VOIs are denoted on d and axial VOIs (sternum, spine, and skull) are highlighted in grey.

Having identified murine bone density to energy metabolism quotient diversity (Figure 2), we tested if individual bones' distinct metabolism formed functional interconnected networks at a system level. A network clustering analysis was performed on the extracted time-activity curves of ^18^F-FDG obtained using dynamic 1-h total-body PET scanning (Figure 3A) to investigate interactions between individual bones and identify if glucose skeletal metabolism networks were present.

We found a unique functional network (Figures 3B,C) whereby there was a high connectivity between long bones (femur, tibia). Meanwhile, the spine showed very little connectivity to any other bony tissue in the glucose metabolism (^18^F-FDG) network. Furthermore, the spine, which had the weakest connectivity in the^18^F-FDG skeletal network, was the only bone to show a strong positive correlation (r^2^ = 0.9965) between ^18^F-FDG uptake and bone density (by CT HU, Figure 3D).

## Discussion

Our study demonstrates that in mice, different bones within the skeleton have unique molecular signatures and form a distinct metabolic network. Of importance, the metabolic dissimilarity observed between the spine and the rest of the skeleton, identified only by the ^18^F-FDG total-body network and not standard whole-body SUV analysis, may be of significant clinical importance and could impact on the development of new treatments for metabolic and bone diseases.

The bones of the skeleton are classically divided into two anatomical classifications: the axial skeleton (bones along the body's long axis) and the appendicular skeleton (appendages of the axial skeleton). In addition to bone location, bones can form *via* two fundamentally different processes. Flat bones (e.g., the skull and scapula) are formed by intramembranous ossification, whereas long bones (e.g., tibia and humerus) are formed by endochondral ossification (15). Traditionally, dual energy x-ray absorptiometry (DEXA) or qCT have been used for quantification of bone density (16, 17). Our data shows that the classification of bones based on anatomical location, formation or density do not recapitulate complex bone metabolic functions as determined by total-body dynamic ^18^F-FDG PET both at individual bone level and system connectivity level. Therefore, highlighting the importance of bone-specific endocrine functions in addition to classic functions in organ protection and locomotion.

Previously, in murine models of ageing, ^18^F-FDG PET/CT analysis has shown that the spine had reduced ^18^F-FDG uptake compared to other skeletal sites and this uptake was reduced with increasing age (12). In humans, osteoporosis, a systemic skeletal disease characterised by low bone mass and microarchitectural deterioration is estimated to be responsible for 80–95% of hip and spine fractures in humans (18, 19). Our total-body PET network finding that the spine has a distinct energy metabolism/bone density dynamics than other bones suggests spine fragility during the ageing process might be underpinned by a stronger dependence on glucose metabolism. This would be supported by evidence showing that altered glucose metabolism commonly occur with ageing (20). It follows from these data that future treatments for bone diseases such as osteoporosis may beneficiate from tailoring of the treatment strategies based on skeletal site-specific metabolic differences while keeping in mind systems level interactions beyond bone mineralisation. The new network approach could help unleash further knowledge on bone function. For example, it could also be interesting to investigate skeletal networks using ^18^F-NaF, a radiotracer previously used as marker of active mineralisation, as metabolic bone disease may be more osteoblastic that osteolytic.

General anaesthesia, typically required for dynamic PET/CT imaging of live rodents, could be a limitation of the current work. Previous studies have shown that isoflurane can affect the uptake of ^18^F-FDG (21, 22), thus it is important to consider this caveat when interpreting *in vivo* small animal PET/CT data.

In conclusion, we have shown that simplistic CT HU and PET SUV analysis fail to interrogate functional system-level networks that are present *in vivo*. Our novel network-based analyses of PET data have highlighted that the spine has a unique glucose metabolic function where bone density is strongly dependent on glucose metabolism. Applying our new PET network analysis approach to other preclinical studies and clinical studies holds great promise in not only revealing further physiological and pathological intricacies of the skeleton, but can also be used to understand physiological and pathological tissue interactions between organ systems. Our data are directly relevant to human health due to the recent development of the first clinical total-body PET systems, which will provide an opportunity to investigate if our findings in mice translate to humans. One can easily envision the application of the innovative total-body PET network analysis technique reported in this paper in a variety of diseases and the characterisation of network changes or losses during pathology, for example, were there is metabolic disruption at system-levels.

## Data Availability Statement

The datasets generated and analyzed for this study will be deposited upon manuscript acceptance in the “PET is Wonderful” data repository hosted by Edinburgh Datashare (https://datashare.ed.ac.uk/handle/10283/3219).

## Ethics Statement

Studies were done in compliance with all relevant ethical regulations under project licences granted by the UK Home Office, and were approved by the University of Edinburgh Animal Welfare and Ethical Review Board.

## Author Contributions

AT and KS: conceptualisation and writing—original draft preparation. KS, CJAC, SN, MGM, RS, CF, TF, and AT: writing—review and editing. AT: funding acquisition. All authors contributed to the article and approved the submitted version.

## Funding

AT is funded by the British Heart Foundation (FS/19/34/34354); and is a recipient of a Wellcome Trust Technology Development Award (221295/Z/20/Z) and a Chan Zuckerberg Initiative DAF grant number 2020-225273, an advised fund of Silicon Valley Community Foundation. KS and RS are funded by the Medical Research Council (MR/S035761/1) and RS is funded by the Chief Scientist Office (SCAF/17/02). MGM is funded by the British Heart Foundation (RG/16/10/32375). CF is supported by the Biotechnology and Biological Sciences Research Council (BBSRC) through an Institute Strategic Programme Grant Funding (BB/J004316/1). The British Heart Foundation is greatly acknowledged for providing funding toward establishment of the preclinical PET/CT laboratory (RE/13/3/30183). We thank Mr. William Mungal for invaluable technical assistance with the animal experiments; CJAC is supported by the Edinburgh Preclinical Imaging core facility.

## Conflict of Interest

The authors declare that the research was conducted in the absence of any commercial or financial relationships that could be construed as a potential conflict of interest.

## Publisher's Note

All claims expressed in this article are solely those of the authors and do not necessarily represent those of their affiliated organizations, or those of the publisher, the editors and the reviewers. Any product that may be evaluated in this article, or claim that may be made by its manufacturer, is not guaranteed or endorsed by the publisher.
